# A179 A CANADA-WIDE STUDY OF TRENDS IN HOSPITALIZATION RATES FOR INFLAMMATORY BOWEL DISEASE

**DOI:** 10.1093/jcag/gwae059.179

**Published:** 2025-02-10

**Authors:** S Coward, E Benchimol, C N Bernstein, A Avina-Zubieta, A Bitton, F Hoentjen, E Kuenzig, N Lu, J Leal, C Ma, S Murthy, K Novak, Z Nugent, A Otley, R Panaccione, J Peña-Sánchez, H Singh, L Targownik, G G Kaplan

**Affiliations:** University of Calgary, Calgary, AB, Canada; The Hospital for Sick Children, Toronto, ON, Canada; University of Manitoba Max Rady College of Medicine, Winnipeg, MB, Canada; The University of British Columbia, Vancouver, BC, Canada; McGill University, Montreal, QC, Canada; University of Alberta, Edmonton, AB, Canada; The Hospital for Sick Children, Toronto, ON, Canada; Arthritis Research Canada, Richmond, BC, Canada; University of Calgary, Calgary, AB, Canada; University of Calgary, Calgary, AB, Canada; University of Ottawa, Ottawa, ON, Canada; University of Calgary, Calgary, AB, Canada; University of Manitoba, Winnipeg, MB, Canada; Dalhousie University, Halifax, NS, Canada; University of Calgary, Calgary, AB, Canada; Department of Community Health and Epidemiology, University of Saskatchewan, Saskatoon, SK, Canada; University of Manitoba Max Rady College of Medicine, Winnipeg, MB, Canada; University of Toronto, Toronto, ON, Canada; University of Calgary, Calgary, AB, Canada

## Abstract

**Background:**

Hospitalizations among individuals with inflammatory bowel disease (IBD) place a strain on healthcare resources. The decline in hospitalization rates during the era of anti-TNF therapies remains debated in the literature.

**Aims:**

To examine temporal trends in hospitalization rates among individuals with in IBD across Canada.

**Methods:**

We used population-based administrative healthcare data (2002–2014) from seven Canadian provinces (AB, BC, MB, NS, QC, ON, SK) to identify hospitalizations in prevalent IBD cases. Hospitalizations were categorized as: 1. all-cause, any hospitalization of an IBD patient; 2. IBD-related, admission for IBD or symptoms/comorbidities associated with IBD (eg. venous thromboembolism). We calculated hospitalization rates per 100 IBD persons with 95% confidence intervals (CIs) using IBD prevalence data. Hospitalization rates were forecast from 2015–2025, with 95% prediction intervals (PIs), using auto regressive integrated moving average models on log transformed data. We calculated average annual percentage change (AAPC) using Poisson models with quadratic equations applied for non-linear trends. We stratified by IBD subtype (CD, UC), age (<18, 18–64, 65+), and sex (female, male). We calculated AAPCs for counts to assess the actual number of hospitalizations.

**Results:**

From 2002–2014, hospitalizations rates decreased for both all-cause and IBD-related admissions for IBD patients, and across age, sex, and IBD type (Table 1). In 2025, we forecast hospitalization rates to be 15.82 (95%CI:14.17,17.66) per 100 for all-cause and 7.87 (95%CI:6.16,9.90) per 100 for IBD-related. Hospitalization rates are falling, but AAPCs for hospitalization counts significantly increased for all-cause (2.65%; 95%CI: 2.42,2.89) and IBD-related (1.52%; 95%CI: 1.29,1.76). The disparity between decreasing rates and increasing counts is due to the faster rise in the AAPC of IBD prevalence (denominator) compared to hospital counts (numerator).

**Conclusions:**

During the anti-TNF era (2002–2014), hospitalization rates for IBD steadily declined across Canada and are projected to continue decreasing through 2025. Despite this decline, the actual number of hospitalizations is increasing, likely driven by the rising prevalence of IBD.

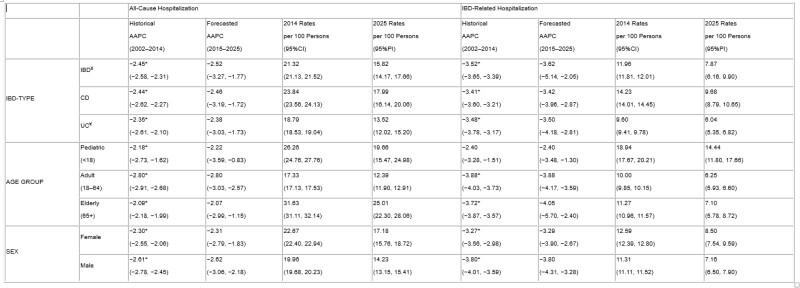

¥ Includes IBD-Unclassified *Non-linear

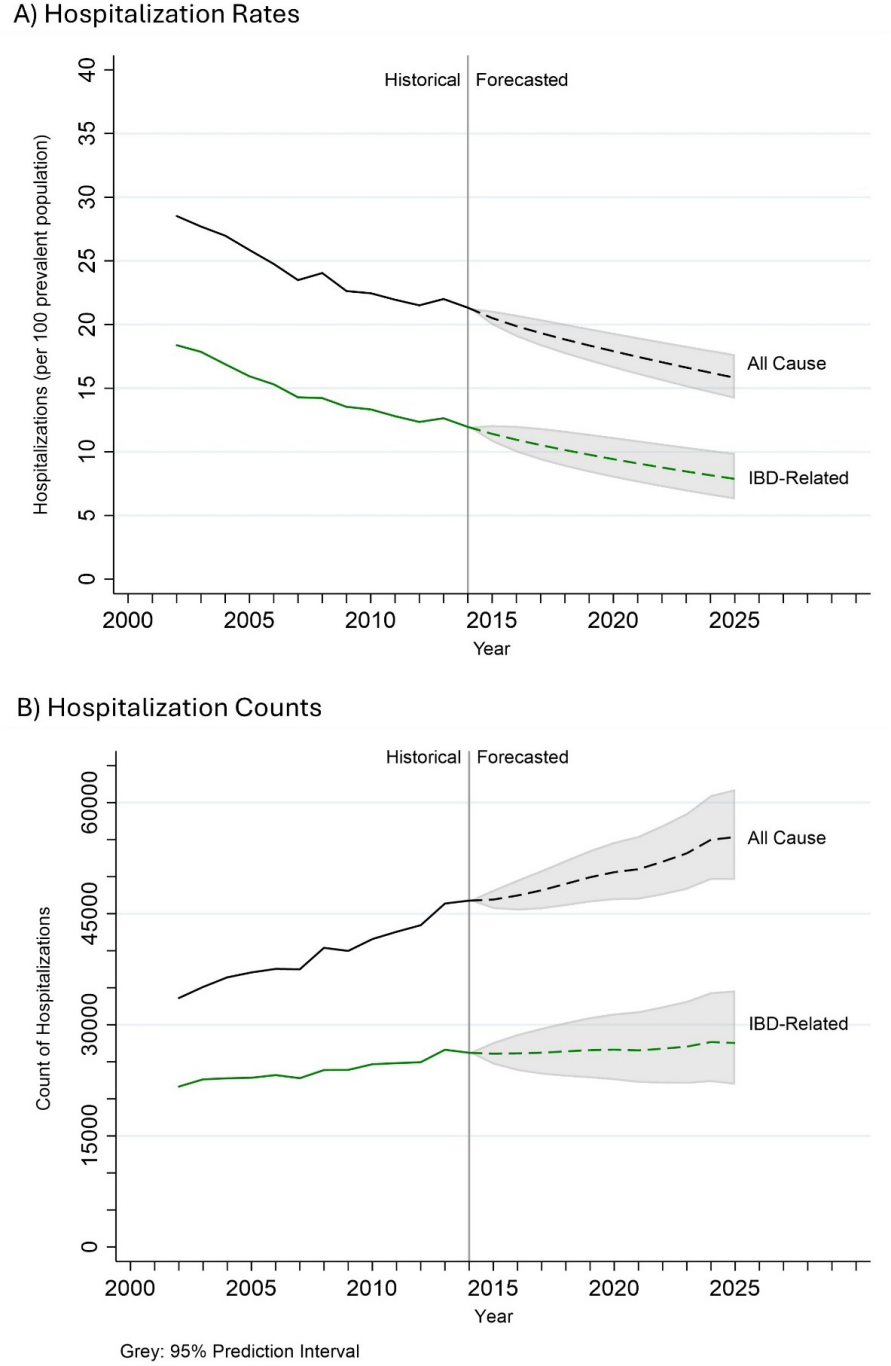

**Funding Agencies:**

CIHR

